# Crosstalk Between Skeletal Muscle and Immune System: Which Roles Do IL-6 and Glutamine Play?

**DOI:** 10.3389/fphys.2020.582258

**Published:** 2020-10-16

**Authors:** Patricia S. Rogeri, Sandro O. Gasparini, Gabriel L. Martins, L. K. F. Costa, Caue C. Araujo, Rebeca Lugaresi, Mariana Kopfler, Antonio H. Lancha

**Affiliations:** Laboratório de Nutrição e Metabolismo, Escola de Educação Física e Esporte da Universidade de São Paulo, EEFE-USP, São Paulo, Brazil

**Keywords:** skeletal muscle, immune system, glutamine, interleukin-6, lymphocytes, macrophages

## Abstract

The skeletal muscle was always seen from biomechanical and biochemical views. It is well-established that an active muscle brings many benefits for different body organs and tissues, including the immune system. Since the 1970s, many studies have shown the importance of regular exercise and physical activity in increasing the body’s ability to fight opportunist infections, as well as a strategy to fight established diseases. This interaction was mainly attributed to the glutamine, a non-essential amino acid produced by the active skeletal muscle and primarily consumed by rapidly dividing cells, including lymphocytes and monocytes/macrophages, as their main source of energy. Therefore, these cells’ function would be significantly improved by the presence of a bigger glutamine pool, facilitating phagocytosis, antigen-presentation, proliferative capacity, cytokine synthesis and release, among other functions. Despite its importance, glutamine is not the only molecule to connect these two tissues. The presence of cytokines is crucial for a proper immune system function. Many of them have well-established pro-inflammatory properties, while others are known for their anti-inflammatory role. Interleukin-6 (IL-6), however, has been in the center of many scientific discussions since it can act as pro- and anti-inflammatory cytokine depending on the tissue that releases it. Skeletal muscle is an essential source of IL-6 with anti-inflammatory properties, regulating the function of the immune cells after tissue injury and the healing process. Therefore, this review aims to discuss further the role of these four components (glutamine, and interleukin-6, and its interface with monocytes/macrophages, and lymphocytes) on the communication between the skeletal muscle and the immune system.

## Introduction

For years, the skeletal muscle was seen from a biomechanical point of view as an organ responsible for producing movement thanks to the contraction of its fibers. Later, the importance of this organ from a biochemical point of view was discovered. It was observed that the skeletal muscle is a crucial energy-consumer tissue when active, consuming glucose and glycogen as essential energy sources, but also consuming the energy accumulated in the adipose tissue, leading to lower body fat percentage, associated with many health and metabolic benefits.

Studies dating back to the 1970s, especially those focused on exercise, have shown the importance of physical activity to the immune system. It has been shown that regular physical activity is essential to increase the organism’s ability to fight opportunist infections, despite an initial, transient exercise-induced immunosuppression (Ahlborg and Ahlborg, 1970; Calder et al., 2007).

For that to happen, a proper metabolic environment plays a pivotal role. The proper plasma concentration of glutamine mainly provides this environment. Glutamine was maybe the first well-established link between the immune system and the skeletal muscle. Under optimal conditions, there is a qualitative and quantitative impact on the immune cells and the muscle itself, improving its ability to secrete proteins known as myokines.

Leukocytes, in general, are sensitive cells affected by exercise. According to Pedersen and Hoffman-Goetz (2000), exercise can induce stress-like responses in the body, similar to those observed after major surgeries, trauma, severe burn, and sepsis. These physical-clinical stressors induce mechanical, metabolic, and hormonal responses to keep body homeostasis. As part of its process, the immune system is affected, with acute and chronic adaptations, leading to adjustments in the inflammatory response and the response of neutrophils, lymphocytes, and monocytes (Pedersen and Hoffman-Goetz, 2000).

Such cells are responsive to hormones, such as adrenaline and cortisol, as well as to cytokines, to keep their constant communication with the skeletal muscle. More recently, proteins similar to the cytokines have been discovered to be produced by the skeletal muscle itself. The so-called myokines, produced by the myocytes and released by muscle contraction during physical exercise, have autocrine, paracrine, and endocrine functions, and help perform the regulation of the immunometabolism thanks to their ability to induce significant metabolic, energetic and hormonal changes (Cannon, 2000; Pillon et al., 2013; Iizuka et al., 2014; Severinsen and Pedersen, 2020).

**FIGURE 1:**
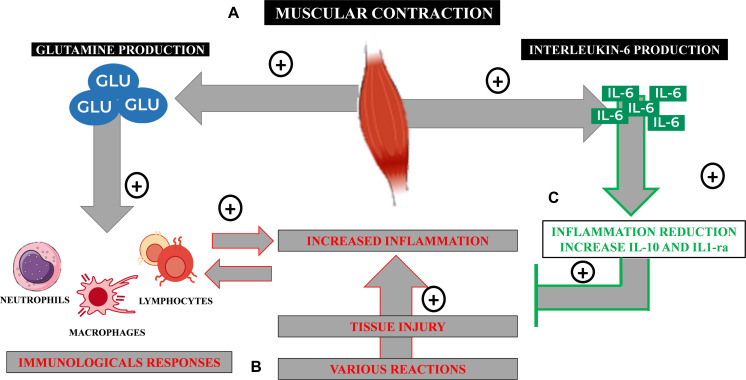
Immunometabolic processes from the practice of physical exercises. **(A)** Glutamine is synthesized by the active skeletal muscle in an ATP-dependent reaction and released from it to the plasma by a bidirectional Nm transportation system. **(B)** Under infectious or inflammatory conditions that lead to tissue injury, an inflammatory reaction takes place activating immune cells, such as neutrophils, macrophages, and lymphocytes. These cells consume large amounts of glutamine to keep their function and immunological performance, including pro-inflammatory cytokines’ synthesis, such as IL-6. **(C)** The skeletal muscle is also capable of producing myokines, such as IL-6, that, in this case, has an anti-inflammatory property, regulating inflammation and assisting on tissue healing processes.

In this review, summarized in Figure 1, we will highlight the important roles of glutamine and interleukin-6 (IL-6), as well as lymphocytes and monocytes/macrophages in the complex communication between the skeletal muscle and the immune system.

## Glutamine

Glutamine is the most abundant free amino acid in the body. It plays a pivotal role in maintaining the function of several organs and cells, such as kidneys, intestines, liver, heart, neurons, leukocytes, and white adipose tissue (Curi et al., 2017; Cruzat et al., 2018). Its production by the skeletal muscle in healthy subjects classifies the glutamine as a non-essential amino acid, however, glutamine concentration varies according to the type of muscle fibers. Type 1 fibers or oxidative fibers can present up to three times more glutamine than type 2 (glycolytic) fibers since type 1 fibers present more glutamine synthetase and more ATP availability than the later (Cruzat and Tirapegui, 2009; Cruzat et al., 2018).

Glutamine may also be considered a conditionally essential amino acid for the amount produced under stressful conditions, such as severe burn, sepsis, infections, major surgeries, and intense exercise, may not be enough to maintain the proper function of the organs and cells previously mentioned (Curi et al., 2017; Soares et al., 2019).

Glutamine is synthesized mainly by the skeletal muscle in an ATP-dependent reaction mediated by glutamine synthetase (GS), which catalyzes it from glutamate and ammonia (glutaminase being the enzyme that catalyzes the reverse reaction, however, it is not found in the skeletal muscle). Glutamine is then released from the muscle and transported to the plasma by a bidirectional N^m^ transportation system affected by glucocorticoids and insulin levels (Walsh et al., 1998; dos Santos et al., 2009).

Glutamine levels increase after intense, short-term exercise and drop after intense, prolonged exercise (Walsh et al., 1998; dos Santos et al., 2009). Although the mechanisms are still under investigation, authors have proposed some mechanisms to explain this phenomenon: high demand by the liver and kidneys for glucose relying on gluconeogenesis to fulfill their demand; increase consumption of glutamine by the immune and other cells; impairment of the mechanisms that promote the release of glutamine by the muscle; and/or a decrease of glutamine synthesis by the muscle (Walsh et al., 1998; dos Santos et al., 2009). In order to shed some light on this subject, dos Santos et al. evaluated different aspects related to the glutamine metabolism: its plasma levels, its transport, GS activity, among others. They used 47 animals distributed in sedentary and trained groups, the later divided into two groups of animals sacrificed 1 h after the last exercise session, and the second sacrificed 24 h after the last exercise session. In possession of plasma and the soleus muscle, the authors observed that glutamine levels were lower in animals sacrificed 1 h after the last exercise session, with a concomitant increase in the corticosterone plasma levels and the GS activity, and lower ammonia levels in the muscle suggesting higher consumption of glutamine by other tissues, such as liver and kidneys. On the other hand, animals sacrificed 24 h after the last exercise session had similar glutamine levels to sedentary animals, with lower plasma levels of corticosterone, lower GS activity, and lower glutamine concentration in the muscle, supporting the lower restoration hypothesis (dos Santos et al., 2009).

Glutamine is an essential fuel for rapidly dividing cells, such as enterocytes, fibroblasts, and leukocytes because it is a precursor of peptides, proteins, nicotinamide adenine dinucleotide phosphate (NADPH), antioxidants, purines, and pyrimidines (Aledo, 2004; Curi et al., 2017). Glutamine also plays an important role regulating the heat shock proteins (HSP) and the reactive oxygen species (ROS), which depending on the intensity and duration of the exercise, can lead to muscle catabolism that contributes to reduce glutamine concentration (Cruzat and Tirapegui, 2009). Therefore, glutamine prepares the physiological environment for these cells’ best function and performance.

When glutamine concentration lowers under one of the stressful conditions mentioned above, cells, such as lymphocytes, macrophages, and neutrophils, have their function and performance impaired due to the lack of their primary source of fuel. Consequently, it is observed lower neutrophils oxidative burst, a decline in some lymphocyte T populations, an impairment of T cell proliferative capacity, and lower macrophages’ phagocytic ability. These conditions lead to immunosuppression, increasing the chances of a person developing infections, such as upper respiratory tract infections (Bassit et al., 2002; Rogeri and Rosa, 2005; Curi et al., 2017; Soares et al., 2019).

Although glutamine production happens primarily by active skeletal muscle, intense muscle contraction increases the demand for glutamine, which competes for the same fuel nutrient as lymphocytes and macrophages, forcing a modulation of these cells in favor of the musculature (Newsholme, 1994). A study with 11 healthy subjects showed that glutamine supplementation was able to increase the glutamine uptake by the skeletal muscle, however, it did not increase the intramuscular concentration of this amino acid, suggesting that there is either a simultaneous increase in the protein synthesis in the tissue or a limit to its accumulation in the muscle (Mittendorfer et al., 2001). Therefore, despite its production, the skeletal muscle also consumes glutamine lowering its availability for other tissues and cells.

During infection, the consumption of glutamine by immune cells is higher than glucose, since glutamine is necessary for T and B lymphocytes proliferation process, as well as for protein synthesis, production of interleukin-2 (IL- 2) and antibody synthesis (Cruzat et al., 2018). Therefore, glutamine metabolism plays a crucial role in lymphocyte activation, and its decline in plasma concentration after intense exercise has been observed (Keast et al., 1995). Also, low levels have been reported as a predictor of overtraining in athletes (Keast et al., 1995). However, the low availability of glutamine cannot be observed in every catabolic or ill patient, and not all individuals benefit from glutamine supplementation. In fact, there is not enough evidence in the literature showing that glutamine supplementation restores immune function after exercise (Keast et al., 1995) and the results of such studies remain controversial and seem to vary according to many factors, such as its form (free or dipeptide) or the association or not with other supplements (Cruzat et al., 2014). A recent study showed that athletes who undergo rapid weight loss for competition purposes, creating significant stress levels to their bodies, did not benefit from glutamine supplementation. The study showed that such athletes present an increased frequency of upper respiratory tract infections in spite of glutamine supplementation, similar to those who received placebo (Tritto et al., 2018).

Rogeri and Rosa (2005) studying spinal cord injured (SCI) people showed that in contrast to healthy subjects, people with that type of injury present a significant decrease in their plasma glutamine concentration. The authors also showed that the higher the injury, which leads to more spread out paralysis throughout the body, the lower the glutamine concentration, with a tendency to increase after a stress test in an adapted treadmill. Their findings suggested that glutamine concentration, and not only mechanical issues suffered by SCI people, is responsible for the high incidence of infection observed in this population when compared to healthy subjects. The authors also suggested that exercise may help restore glutamine concentration (Rogeri and Rosa, 2005).

Due to its importance to the immune system, glutamine became very popular and was consumed by many people as an attempt to improve their immune response. In the late 1980s and early 1990s, studies in animals have shown that most of the glutamine orally consumed would not enter the bloodstream but instead remained in the intestinal lumen, being consumed by enterocytes (Newsholme, 1994). Therefore, studies have shown that a more efficient way to obtain positive results on the immune system is by consuming glutamine precursors, such as branched-chain amino acids (BCAA) (Bassit et al., 2000, 2002). Bassit et al. (2000, 2002) showed in two different studies that athletes that consumed placebo had a lower plasma glutamine concentration after their exercise session that was reverted by BCAA consumption with a consequent increase in the immune cells’ proliferative capacity and cytokine modulation. Although most amino acids are metabolized in the liver, this organ possesses low BCAA aminotransferase activity, causing the BCAAs to be metabolized primarily in the skeletal muscle (Walsh et al., 1998).

Finally, glutamine degradation into glutamate, in a reaction catalyzed by glutaminase, as previously mentioned, provides an important precursor to glutathione synthesis, the most abundant non-protein thiol in the body. It acts as a powerful antioxidant, working in the xenobiotic detoxification, regulating essential cell functions such as proliferation and apoptosis, and acting upon the immune function and fibrogenesis. Therefore, the glutathione has a pivotal role in protecting the mitochondria against physiological and pathological stressors created by the ROS (Lu, 2013; Draganidis et al., 2016).

## Interleukin-6

Interleukins are cytokines that trigger diverse immunomodulatory functions after changes in their physiological levels, which may induce pro-inflammatory, anti-inflammatory, or even both effects, according to the organism and/or the cell group in which they are synthesized (Brocker et al., 2010).

In this context, Interleukin-6 (IL-6) is the cytokine that shows the highest plasma elevations after acute physical exercise (Febbraio and Pedersen, 2002), with its plasma peak being directly influenced by the intensity (Leggate et al., 2010), the daily frequency (Ronsen et al., 2002), and/or the duration (Fischer, 2006) of the proposed exercise. It is currently proposed that the increase in IL-6, from muscle contraction, can trigger positive effects not only on muscle tissue but also on bone and mitochondrial health, and the control of low-grade chronic inflammation, through IL-6 anti-inflammatory effects in parallel with its performance in lipid oxidation (Fix et al., 2019; Wedell-Neergaard et al., 2019; Cornish et al., 2020).

In contrast to the acute elevations of IL-6 after physical exercise, the literature demonstrates that the improvement of physical conditioning in different populations is strongly associated with lower baseline (resting state) plasma values of IL-6 (Cesari et al., 2004; Colbert et al., 2004; Panagiotakos et al., 2005; Bruun et al., 2006). It suggests that increased levels of IL-6 in the absence of exercise may be directly related to a higher degree of physical inactivity and metabolic syndrome (Bruun et al., 2006; Fischer et al., 2007). This “contradictory action” of IL-6 occurs because this interleukin can be produced not only by the immune system cells, but also by different tissues such as adipocytes (Coppack, 2001; Lyngsø et al., 2002), and muscle fibers through the initial infiltration of macrophages in the muscle tissue (Tominaga et al., 2019) or by the subsequent production of this interleukin by myoblasts (Gallucci et al., 1998; Pillon et al., 2013), thus triggering systemic pro-inflammatory (Coppack, 2001) or anti-inflammatory (Gallucci et al., 1998; Pillon et al., 2013) effects, respectively.

Despite lacking recent original studies characterizing the biomolecular mechanisms behind the elevations of IL-6 in muscle fibers, some synergistic action between the infiltration of immune cells mediated by the practice of physical exercise in muscle tissue has been proposed as a determining factor for the regulation of muscle damage and inflammation (Gallucci et al., 1998; Kawanishi et al., 2016; Tominaga et al., 2019). In this context, the total plasma level of IL-6 can also be partially altered by immunological cells from the innate immune system (such as macrophages and neutrophils), as well as from the adaptive immune system (such as T and B cells) (Nielsen et al., 1996), depending on the training stimulus performed, ideally inducing transient pro-inflammatory effects, with a posterior anti-inflammatory response (Kawanishi et al., 2016).

From this perspective, the metabolic pathways, which can trigger substantial increases in IL-6 in muscle tissue, mediated by the regular practice of exercise, exert their effects locally on muscle cells, favoring more pronounced increases in IL-6 in the tissue, through a homodimer gp130Rb/IL-6Ra, which results in the activation of AMPK and/or phosphatidylinositol 3-kinase (PI3-kinase) (Pedersen and Fischer, 2007). In this sense, reduced concentration of muscle glycogen, previously or after the practice of exhaustive aerobic exercises, is considered an essential factor that favors the marked appearance of IL-6 in plasma via AMPK activation in myoblasts (Bartoccioni et al., 1994; Pedersen and Febbraio, 2008). This pathway can trigger more significant bioenergetic changes from the acute increase in IL-6. Also, it is proposed that this increase in plasma IL-6 levels may be partially influenced by the increased release of ionic calcium from the muscle sarcoplasmic reticulum, stimulating the activation of the nuclear factor of T cells (via calcineurin), which is present in the muscle (Bartoccioni et al., 1994; Holmes et al., 2004). In these two metabolic pathways, it has been shown that the produced IL-6 provides anti-inflammatory effects in the body, inhibiting, for example, endotoxin mediated by substantial increases in TNF-alpha levels in humans (Starkie et al., 2003; Keller et al., 2006; Hennigar et al., 2017), in addition to inducing the subsequent release of other cytokines with anti-inflammatory function [interleukin-1 receptor antagonist (IL-1ra) and interleukin-10 (IL-10)] (Hotamisligil et al., 1996).

Currently, these physiological actions were considered relevant to indicate that the increase in IL-6, mediated by physical exercise, can trigger positive reflexes on the individual’s insulin sensitivity (Steensberg, 2003), in addition to the increase in lipid oxidation (Carey et al., 2006), without performing “undesirable” pro-inflammatory effects to the proper functioning of the immune energy metabolism. Also, increases in IL-6 with an anti-inflammatory characteristic have been the target of encouraging studies involving a possible therapeutic effect of IL-6 in chronic diseases that establish a chronic environment of low-grade inflammation, such as arthritis rheumatoid (Carey et al., 2006), sarcopenia (Beyer et al., 2012) and even cancer (Daou, 2020). In this sense, although it is not completely clear, IL-6 seems to be involved in immune metabolic issues from its production in myocytes and immune cells, during and immediately after the exercise.

In a recent study, Wedell-Neergaard et al. (2019) investigated the peripheral effects of IL-6. Fifty-three subjects with high central adiposity performed 12 weeks of aerobic training (intensities ranging from 50 to 85% of VO2max) with or without the presence of an IL-6 receptor blocker (tocilizumab). In the study (Wedell-Neergaard et al., 2019), the group that performed the training sessions with the administration of tocilizumab showed significant lower reductions in visceral fat compared to the group trained without administration of tocilizumab, with no lean mass and subcutaneous fat tissue difference. As mentioned by the authors “as visceral adipose tissue was found to express more IL-6 receptors than subcutaneous adipose tissue, it is most likely that visceral adipose tissue is more sensitive and responsive to changes in IL-6 than subcutaneous adipose tissue” (Wedell-Neergaard et al., 2019), indicating that not only the increase but also the action of IL-6 are strictly related to the visceral fat reduction in humans. Since central adiposity is associated with an increase in low-grade chronic inflammation, regardless of BMI (Wedell-Neergaard et al., 2018), it is relevant that future studies aim to clarify the mechanisms by which the acute increase in IL-6, in the context of exercise, could impact the mobilization of body fat deposits as well as its relationship with the recruitment of specific immune cells between different populations.

## Lymphocytes

Like most tissues, skeletal muscle contains a resident population and additional infiltrate immune cells during pathophysiological conditions, such as reperfusion-induced contraction or injury, endotoxemia, or inflammatory myopathies, due to the action of cytokines or factors with attractive properties and activation (Pillon et al., 2013).

Many studies have shown that exercise induces a short period of leukocytosis followed by another period of leukopenia, when mainly T cells suffer a significant decrease in its population, creating an opportunity for opportunistic infections to occur. According to Nieman (1994), immunological changes would be accentuated as the intensity increases, theory postulated on his famous “J” curve to explain the relationship between exercise intensity and risk of upper respiratory tract infections (URTI) (Nieman, 1994).

Intensity and duration of physical effort would be determinant to the proliferative response of T lymphocytes (Shinkai et al., 1992), as observed by a more significant increase in plasma cytokine levels at high intensities (Berk et al., 1989; Keast et al., 1995). In response to IL-2 released during intense muscle contraction, more natural killer (NK) cells, monocytes, and B cells are attracted due to their prominent responsiveness than any other subpopulation (Pedersen and Hoffman-Goetz, 2000), causing the relative decline of TCD4++ cells percentage (Fry et al., 1992).

Lymphocytes concentration decrease in the post-exercise period has also been associated with an apoptosis mechanism induced by exercise (Navalta et al., 2007) and more observed with a gradual increase in intensity, reaching the maximum peak immediately after exhaustive exercise (100% VO_2_max), in percentages of apoptosis around 22% (Steensberg et al., 2002). There are reports about 63% of lymphocytes apoptosis after high intensity (Mars et al., 1998). Some authors tend to associate the phenomenon to action from high levels of catecholamines (Navalta et al., 2007), able to decrease the concentration of lymphocyte glutathione and increase oxidative stress (Wang and Huang, 2005) and the production of ROS, in addition to increased fragmentation of DNA (Mooren et al., 2002). However, the mechanism responsible for post-exercise apoptosis remains to be elucidated by science. In this meantime, researchers debate whether exercise could contribute to the marked apoptosis of lymphocytes, and criticize studies based on different sampling time, lack of methodologies standardization, and some subsets lymphocytes absence (Simpson et al., 2007; Navalta et al., 2010).

Overload during exercise causes microtrauma of varying degrees in muscle tissue that are considered temporary and repairable damage by the immune system, activated immediately after the injury by cellular debris and leakage of the cellular content from damaged fibers. Muscle contraction itself increases calcium and pro-inflammatory cytokines release, such as tumor necrosis factor-alpha (TNF-α) and interleukin-1 beta (IL-1β), which together sarcolemma lesion and eicosanoids derived release (Smith, 2004) from the constituents from arachidonic acid of cell membranes, attract neutrophils, monocytes, lymphocytes and other cells to the injured site generating acute inflammatory response (Smith, 2000) and initiating cleaning and indirectly signalizing diapedesis (Moldoveanu et al., 2001), that is, the influx of cells to the site, vasodilation regulation, chemotactic activity and increase in permeability of the vascular endothelium (Tidball, 2005).

Both innate and adaptive immune systems are activated after muscle injury. However, their cells are recruited in an orderly manner to make the environment more conducive to each phase of regeneration. In a first pro-inflammatory moment, debris is cleared, and satellite cells are activated. T cells are removed to the lymphatic system mediated by the action of cortisol (Deyhle and Hyldahl, 2018), perhaps to avoid the potential risk of self-recognition of intracellular debris by the adaptive system, explaining how acute exercise does not redistribute T and B cells in the circulation in the same extent as other cells of innate system. Additionally, lactate production or increased acidity may impact leukocyte redistribution, associated with a higher catecholaminergic response that may also play a role in modifying this cell redistribution (Freidenreich and Volek, 2012).

Macrophages phagocyte the undesirable elements produced by tissue damage (Tidball, 2005). At the same time, IL-6 and interleukin-8 (IL-8) secreted after the damage stimulate the signaling pathway that activates NADPH-oxidase in the process known as respiratory burst, culminating in the release of ROSs (Brickson et al., 2001), chemokines, prostaglandins, hormones such as insulin-like growth factor and some cell growth-regulating cytokines, such as transforming growth factor beta-1 (TGF-β1), which activate fibroblasts to secrete collagen molecules for tissue regeneration, in addition to activating satellite cells for restructuring tissue (Pedersen et al., 1998). This acute inflammatory response must be very well regulated to preserve the integrity of adjacent cells and tissues, avoiding exacerbating damage by exaggerating ROS production (Tidball, 2005). The balance between the pro and anti-inflammatory actions of different cytokines, controlled by an intrinsic program of satellite cells or modulated by extrinsic cells, such as eosinophils and T cells, contributes to the complete regeneration of damaged tissue (Petersen and Pedersen, 2005; Schiaffino et al., 2017).

In a second moment, T cells are recruited to convert the environment into anti-inflammatory and allow the expansion and differentiation of satellite cells and maturation of newly formed microfibers. M1 macrophages attract them about 3 days after the injury starts. They become involved in repairing the skeletal muscle by secreting a variety of growth factors and cytokines that modulate the microenvironment of inflammation. Similar to macrophages, T cells secrete growth factors and cytokines such as TNF-α, interferon gamma (IFN-γ), IL-1β, interleukin-4 (IL-4), interleukin-12 (IL-12), interleukin-13 (IL-13), which modulate the microenvironment to make it more conducive to muscle regeneration, raising the hypothesis that the inflammatory environment could activate and improve the functions of satellite cells (Yang and Hu, 2018).

T regulatory (Treg) cells are important controllers of immune tolerance and accumulate a few days after the injury, attracted by interleukin-33 (IL-33) concentration, a nuclear cytokine released during cell necrosis or tissue damage (Nascimento et al., 2017). In addition to regulating the cells directly responsible for repairing injured muscle, Treg also acts directly on tissue regeneration through the proliferation of muscle satellite cells, releasing amphiregulin, the main autocrine growth factor for human keratinocyte culture and a well-known promoter of tissue healing and regeneration (Burzyn et al., 2013).

Tregs can control inflammation by restricting the immune responses of other cells, both modulation of CD4, CD8 [via the release of inhibitory cytokines such as IL-10, TGF-β, and interleukin-35 (IL-35)] and NK cells (Panduro et al., 2018), controlling the behavior of neutrophils.

Tregs promote environment conversion from pro to anti-inflammatory by releasing anti-inflammatory cytokines (for example, IL-4, IL-10, IL-13) that stimulate M1 (bactericidal and inflammatory) to M2 (immunomodulatory) macrophages phenotype exchange, apoptosis or inhibition of neutrophil inflammatory activity (Li et al., 2018).

## Monocytes/Macrophages

After a muscle injury, an inflammatory response very well organized begins, leading to activation and differentiation of a variety of tissue and immune cells, aiming to repair the injury, leading to a complete recovery of the skeletal muscle (Cohen and Mosser, 2013; Peake et al., 2017).

After tissue injury, specific molecules known as chemotactic mediators are released to the bloodstream attracting monocytes, circulating cells from the immune system responsible for initiating, with neutrophils, the inflammation process, and tissue repair (Contrepois et al., 2020). Monocytes are heterogeneous cells, exhibiting specific functions, and are differentiated by their size, immune receptor expression, and proliferative capacity (Taylor et al., 2005; Shi and Pamer, 2011). They can be classified in three subtypes based on their cluster of differentiation, CD14 and CD16 (Strauss-Ayali et al., 2007) in classic monocytes (CD14++/CD16-) with phagocytic function and that also express genes involved to angiogenesis, wound healing, and coagulation (Hallam and Hagemann, 2012; Yang et al., 2014); non-classical pro-inflammatory monocytes (CD14+/CD16++) responsible for patrolling the tissues (Strauss-Ayali et al., 2007); and intermediate monocytes CD14++/CD16+ or pro-inflammatory monocytes (Hallam and Hagemann, 2012; Yang et al., 2014).

When tissue damage happens, monocytes migrate to the injured area and attach themselves to the extracellular matrix. Some components of the matrix, such as fibrinogen and collagen, seem to stimulate macrophage phagocytosis and pro-inflammatory factors expression (Dort et al., 2019).

The acute inflammatory response after tissue damage begins with neutrophil (Schneider and Tiidus, 2007; Kawanishi et al., 2010), followed by macrophage infiltration. The later acquire particular features depending on the microenvironment they attach to Kosmac et al. (2018). Macrophages represent the biggest pool of cells recruited to the skeletal muscle after injury and play a unique role in regulating the inflammatory process and tissue repair (Wang et al., 2014). Therefore, the recovery of the damaged tissue depends on the macrophage presence and action (Perandini et al., 2018).

In an experimental study with rats, Dort et al. (2019) observed that monocytes expressing Ly6Chi (with phagocytic and pro-inflammatory properties) secrete pro-inflammatory cytokines that attract more neutrophils and monocytes to the site of injury. This pro-inflammatory environment lasts for 48 h after the tissue damage. After that period, monocytes Ly6Clo, responsible for tissue repair, become more predominant, reducing the inflammatory process (Dort et al., 2019).

Once monocytes become resident cells, they also express different phenotypes depending on their activation state (Lee et al., 2020). They co-express CD11b and CD206 and participate in tissue repair by secreting chemotactic factors, having low phagocytic property (Dort et al., 2019). According to their immune function, resident macrophages can be classified in M1 or M2 macrophages (Dort et al., 2019). M1, or classically activated macrophages, have an overall pro-inflammatory behavior, secreting different cytokines, such as TNF-α, interleukin-1 alfa (IL-1α), monocyte chemoattractant protein 1 (MCP-1), monocyte chemotactic protein 3 (MCP-3), macrophage inflammatory protein 2 (MIP-2), oncostatin M (OSM), and vascular endothelial growth factor (VEGF). They also express high inducible nitric oxide synthase (iNOS) activity with a consequent increase in the ROS. M2 macrophages can be divided into three subsets, each one depending on a specific polarization signal. IL-4 and IL-13 exposure activates M2a macrophages, while M2b polarization happens through Il-1 receptor ligands, and M2c polarization is promoted by IL-10 and glucocorticoids (Dort et al., 2019; Lee et al., 2020). M2 macrophages are responsible for regulating the tissue repair process (Dort et al., 2019; Wang et al., 2014). In fact, M1 and M2 act in a perfect balance and together are responsible for the skeletal muscle homeostasis (Lee et al., 2020).

Lemos et al. (2015) showed that skeletal muscle resident M1 and M2 macrophage-produced cytokines modulate the extracellular matrix production through the fibro/adipogenic progenitor cells (FAPs). It has been shown that the production of extracellular matrix components by the FAPs is regulated by TNF-α and by the TGF-β1) secreted, respectively, by M1 and M2 macrophages. The kinetics between M1 and M2 macrophages after a skeletal muscle injury promotes FAPs apoptosis, avoiding an excessive extracellular matrix deposition on the tissue, and an inefficient regeneration process (Mann et al., 2011; Lemos et al., 2015). Based on these findings, FAPs and macrophages were characterized as part of the cells associated with a favorable microenvironment responsible for the activation and differentiation of satellite cells during the skeletal muscle repair process (Jonsdottir et al., 2000; Mashinchian et al., 2018).

After the skeletal muscle injury, an increase in the number of FAPs for the first 1–3 days starts, and it reduces between days 4 and 7 after injury (Lemos et al., 2015). This initial increase on the FAPs is essential for the production of the extracellular matrix components in order to stabilize the tissue, acting as a scaffolding for new fibers, being used by the satellite cells as a basal membrane to assure that the myofibers will remain aligned (Chen and Li, 2009; Mann et al., 2011; Lemos et al., 2015; Munoz-Canoves and Serrano, 2015). This process must be tightly regulated, and the FAPs decline is essential to prevent excessive extracellular matrix deposition, impairing tissue regeneration (Mann et al., 2011).

FAPs kinetics is modulated by cytokines produced and released by both pro- and anti-inflammatory macrophages. A study showed that TNF-α leads to a significant decrease in FAPs after a skeletal muscle injury and that the primary source of this cytokine is the pro-inflammatory macrophages, showing the importance of this joint work between FAPs and macrophages to avoid excessive extracellular matrix deposition (Lemos et al., 2015). These findings were corroborated by studies that attempted to treat pulmonary fibrosis with an anti-TNF monoclonal antibody, which caused pathological accumulation of extracellular matrix (63.64). Plus, pro-inflammatory macrophages increased is followed by anti-inflammatory macrophages during skeletal muscle tissue repair, increasing the expression of TGF-β, which blocks FAPs’ TNF-α-induced apoptosis (Arnold et al., 2007).

While acute inflammatory response is associated to a proper skeletal muscle tissue repair and regeneration, chronic, non-decisive inflammation, such as those observed in pathological conditions like idiopathic inflammatory myopathies, dystrophies, and obesity are associated to impaired satellite, immune and FAP cells function, leading to increased fibrosis and weak muscle regeneration (Keller et al., 2003; Villalta et al., 2009; Kawanishi et al., 2010; Mann et al., 2011; Kong et al., 2013; Lemos et al., 2015). A consistent imbalance between pro- and anti-inflammatory macrophages in the skeletal muscle is associated with impaired satellite cell differentiation and activation (Li et al., 2003; Villalta et al., 2009). Also, chronic inflammation leads to an excess of cytokines responsible for extracellular matrix production (Perandini et al., 2018), causing pro-fibrotic components accumulation, and therefore a non-favorable environment for proper muscle tissue repair (Villalta et al., 2009; Lemos et al., 2015).

Therapies aiming to reduce inflammation and muscle fibrosis have been developed with both beneficial and side effects (Li et al., 2003; Andreetta et al., 2006; Villalta et al., 2009). In an experimental model, it was observed that TGF-β inhibition reduces connective tissue and fibrosis in mice diaphragm but is followed by an increased inflammatory process (Andreetta et al., 2006). Also, some therapies attempted so far cause an imbalance between M1 and M2 cells, preventing the proper establishment of an environment that would allow satellite and other cells involved in the regeneration process to respond optimally (Li et al., 2003; Villalta et al., 2009).

Experimental studies showed that exercise could alter resident macrophages’ phagocytosis, chemotaxis, and antigen presentation capacity abilities, indicating that physical activity can affect these cells function and phenotype (Kawanishi et al., 2010; Walsh et al., 2011).

A recent study showed that resident macrophages are capable of self-regeneration, are kept virtually the same up to adulthood, and respond to small attacks without monocyte infiltration (Davies et al., 2013). The skeletal muscle in response to exercise secretes protons, lactate, ATP, and other factors capable of directly activate macrophages and change their phenotype in response to stimuli (Jonsdottir et al., 2000; Keller et al., 2003; Li et al., 2003). For example, ATP increases pro-inflammatory cytokine release, promoting the expression of more M2 cells, while protons will increase endocytosis and IL-10 secretion by macrophages (Cohen and Mosser, 2013; Cohen et al., 2013; Kong et al., 2013).

Leung et al. (2016) showed a significant increase in the proportion between classic and regulatory macrophages after long-term exercise, suggesting that exercise can help the cells to transition between a pro-inflammatory to an anti-inflammatory state, or even create a mixed phenotype with characteristics both pro- and anti-inflammatory, which could be a protective factor against chronic pain after long or intense exercise sessions (Leung et al., 2016).

## Conclusion

The communication between the skeletal muscle and the immune system happens in many different ways and involves different aspects. Glutamine, a non-essential amino acid, seems to be strongly present in this communication. It is produced by the skeletal muscle, and is used as an energy source by leukocytes, mainly monocytes, and lymphocytes, but also is consumed by the muscle under intense contraction (Keast et al., 1995). In fact, its plasma level has been established as a marker for exercise severity (Keast et al., 1995). Studies have shown the importance of a healthy, in constant contraction skeletal muscle to keep glutamine at optimal levels to assist the immune response (Rogeri and Rosa, 2005). In some conditions, glutamine may not be adequately produced by the muscle, turning it into a conditionally essential amino acid (Curi et al., 2017; Cruzat et al., 2018). When glutamine concentration lowers under stressful conditions, cells such as lymphocytes, macrophages, and neutrophils have their function and performance impaired due to the lack of their primary source of fuel (Bassit et al., 2002; Rogeri and Rosa, 2005; Curi et al., 2017; Soares et al., 2019). A condition that can affect the immune system response due to the lack of glutamine is intense muscle contraction. In this situation, the glutamine produced is used by the muscle itself, turning the organ a competitor with the immune system for this critical substrate (Newsholme, 1994).

IL-6 also plays an essential role in this communication and is the interleukin that shows higher plasma levels after physical exercise (Febbraio and Pedersen, 2002). It can be produced both by the immune system and the skeletal muscle, with pro- and anti-inflammatory properties, respectively (Nielsen et al., 1996). This unique characteristic is important to modulate how immune cells will behave during tissue healing and repair.

**FIGURE 2:**
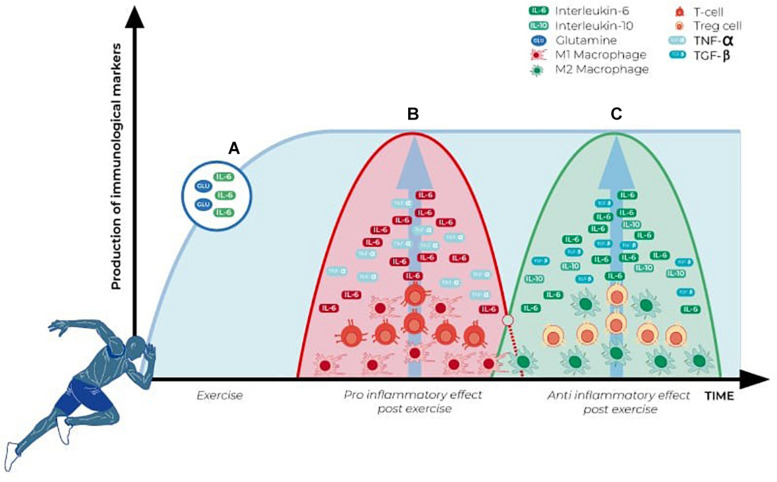
Alteration in immune parameters associated with physical exercise and skeletal muscle activation. **(A)** Glutamine and IL-6 (myokine) levels increase during physical activity and remain elevated in the first moments after it ceases. **(B)** Pro-inflammatory wave initiated immediately after the exercise, with a significant increase in CD4^+^ cells, M1 macrophages, and pro-inflammatory cytokines (TNF-α and IL-6). **(C)** Anti-inflammatory wave induced as a final response after the exercise-induced immune alterations, characterized by a significant increase in the anti-inflammatory cytokines (TGF-β, IL-10), myokines (IL-6), and activation of T regulatory cells and M2 macrophages.

Even under less intense exercise, the skeletal muscle suffers microlesions, and an inflammatory response takes place to solve it (Hedayatpour and Falla, 2015; Hody et al., 2019). This inflammatory response attracts immune cells from the circulation, while tissue-resident cells are activated. As Figure 2 shows, this response happens in distinctive phases. At first, there is an elevation of glutamine and IL-6 to prepare the environment and provide substrates and chemotactic factors for the immune cells. In response to this microinjury, T cells are recruited while M1 resident macrophages are activated, initiating a first, pro-inflammatory wave (Yang and Hu, 2018). Lymphocytes behave in a biphasic pattern: the lymphocytosis observed right after the injury is followed by lymphocytopenia (Toft et al., 1992; Tidball, 2005; Freidenreich and Volek, 2012). However, these findings are mostly related to CD4+ lymphocytes. What happens is the removal of CD4+ cells from the area and an increase in T regulatory cells. These cells act with M2 macrophage cells from the tissue to control the inflammatory response, promoting a more efficient tissue repair, avoiding extracellular matrix excess and fibrosis (Mann et al., 2011; Lemos et al., 2015; Mashinchian et al., 2018; Yang and Hu, 2018).

Therefore, the communication between the skeletal muscle and the immune system seems to be very intense, finely tuned, and dependent on many different factors, such as the ones described above. The tight balance among them provides a proper environment not only for the skeletal muscle repair but also to improve immune system function and responsiveness.

## Author Contributions

PR and AL conceived the present idea. SG, GM, LC, CA, RL, and MK developed the theory. GM created the image. All authors discussed it and contributed to the final manuscript.

## Conflict of Interest

The authors declare that the research was conducted in the absence of any commercial or financial relationships that could be construed as a potential conflict of interest.
